# Combining the best interest standard with shared decision-making in paediatrics—introducing the shared optimum approach based on a qualitative study

**DOI:** 10.1007/s00431-020-03756-8

**Published:** 2020-08-18

**Authors:** Jürg Caspar Streuli, James Anderson, Sierra Alef-Defoe, Eva Bergsträsser, Jovana Jucker, Stephanie Meyer, Sophia Chaksad-Weiland, Effy Vayena

**Affiliations:** 1 Institute of Biomedical Ethics and History, University of Zurich, Winterthurerstrasse 30, 8006 Zurich, Switzerland; 2 University Children’s Hospital and Children’s Research Center, Zurich, Switzerland; 3 Department of Bioethics, The Hospital for Sick Children, Toronto, Canada; 4 Department of Health Sciences and Technology, ETH Zurich, Zurich, Switzerland

**Keywords:** Paediatric ethics, Parental authority, Best interest standard, Qualitative research, Shared decision-making

## Abstract

Paediatric decision-making is the art of respecting the interests of child and family with due regard for evidence, values and beliefs, reconciled using two important but potentially conflicting concepts: best interest standard (BIS) and shared decision-making (SD-M). We combine qualitative research, our own data and the normative framework of the United Nations Convention on the Rights of Children (UNCRC) to revisit current theoretical debate on the interrelationship of BIS and SD-M. Three cohorts of child, parent and health care professional interviewees (*N*_total_ = 47) from Switzerland and the United States considered SD-M an essential part of the BIS. Their responses combined with the UNCRC text to generate a coherent framework which we term the shared optimum approach (SOA) combining BIS and SD-M. The SOA separates different tasks (limiting harm, showing respect, defining choices and implementing plans) into distinct dimensions and steps, based on the principles of participation, provision and protection. The results of our empirical study call into question reductive approaches to the BIS, as well as other stand-alone decision-making concepts such as the harm principle or zone of parental discretion.

*Conclusion*: Our empirical study shows that the BIS includes a well-founded harm threshold combined with contextual information based on SD-M. We propose reconciling BIS and SD-M within the SOA as we believe this will improve paediatric decision-making.

**Table:** 

**What is Known:** *• Parents have wide discretion in deciding for their child in everyday life, while far-reaching treatment decisions should align with the child’s best interest.* *• Shared decision-making harbours potential conflict between parental authority and a child’s best interest.* **What is New:** *• The best interest standard should not be used narrowly as a way of saying “Yes” or “No” to a specific action, but rather in a coherent framework and process which we term the shared optimum approach.* *• By supporting this child-centred and family-oriented process, shared decision-making becomes crucial in implementing the best interest standard.*

**Electronic supplementary material:**

The online version of this article (10.1007/s00431-020-03756-8) contains supplementary material, which is available to authorized users.

## Introduction

The best interest standard (BIS) and shared decision-making (SD-M) feature widely but mostly independently in paediatric reasoning [1–4]. Each has attracted criticism, usually due to misunderstandings around “best” or “shared” [3, 5, 6]. While the BIS is a (contested) ethico-legal standard for determining acceptable options by limiting the authority of designated decision-makers, SD-M is the process by which patients, parents and health care professionals (HCPs) share the best available evidence for informing preferences [4, 7, 8].

The BIS has the advantage of being an individually definable concept based on United Nations children’s rights (Article 3.1, United Nations Convention on the Rights of Children [UNCRC]) and national legislation [9]. The UNCRC respects children’s rights and ensures that special needs for their protection and development are met, summarised in the three Ps: protection, provision and participation. It is not just about the “best” possible objective medical interest, as illustrated by the conflicting parental and HCP viewpoints in the recent Charlie Gard case [3, 10, 11]. Critics of the BIS either reduce it to a single principle such as Diekema’s “harm standard” [12] or prioritise family and parental discretion [13, 14], while its advocates view it as a multistep umbrella concept for reconciling “best” with “harm” [15, 16].

Meanwhile SD-M has been playing an increasing role in paediatric practice. Birchley, however, concluded that it conflicts with the BIS because it is unable to protect the child’s interests [1]. But no study has considered a combination of both.

From 2010 to 2018 we conducted and analysed qualitative interviews and focus groups with patients, parents and HCPs. Our purpose was to study the role of BIS and SD-M in paediatric decision-making.

## Methods

**Fig. 1:**
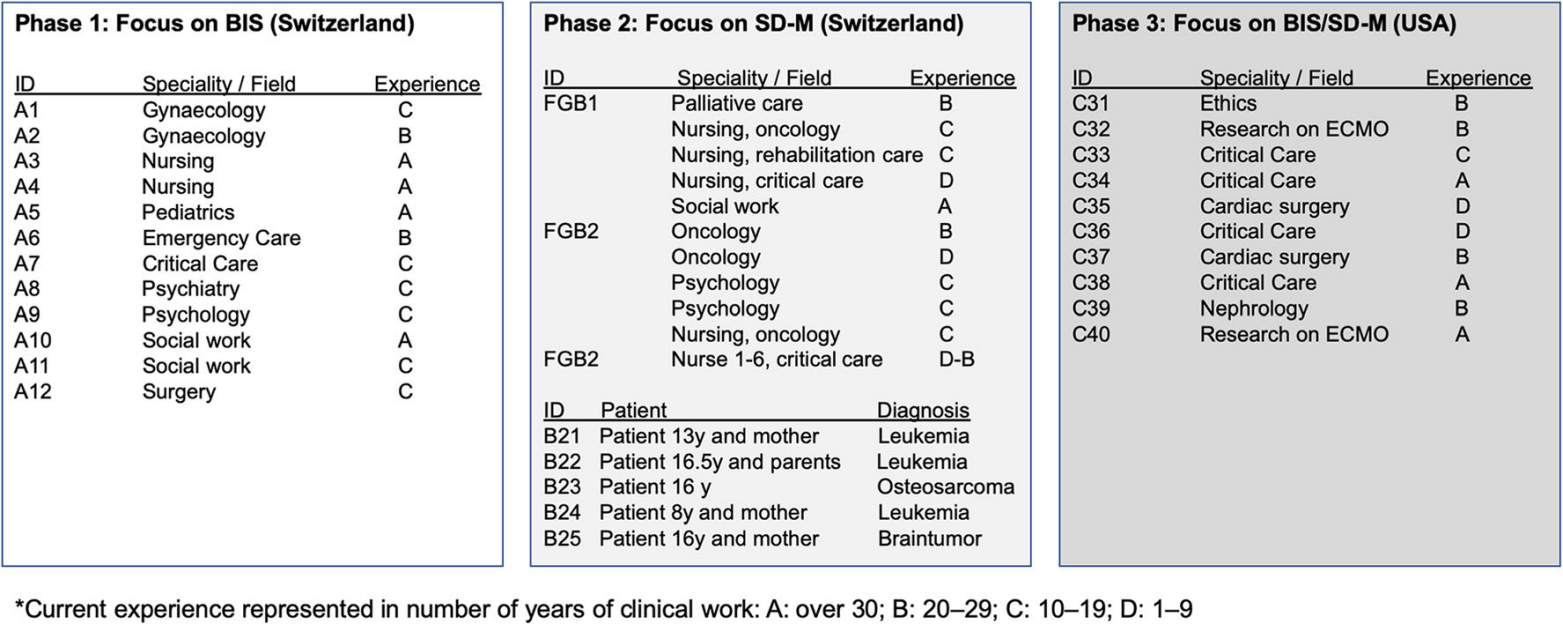
Participants in the three phases. BIS, best interest standard; SD-M, shared decision-making

We obtained our qualitative data from three consecutive subprojects that ran from 2010 to 2018 (Fig. 1), described in detail using the Consolidated Criteria for Reporting Qualitative Studies (COREQ) 32-item checklist (S1) [17]. All participants provided informed consent. We conducted 17 interviews and three focus groups in German (phases I and II) and 10 in English (phase III) (*N*_total_ = 47).

Phase I focusing on BIS and its clinical implementation was conducted with HCPs from the child protection service in a Swiss teaching hospital. Interviews began with open questions on the BIS using hypothetical decision-making scenarios modified and anonymised from former ethical talks [18] (supplement S4). German “Kindeswohl” and English “best interest” were used simultaneously and synonymously (e.g. “How do you define Kindeswohl/best interest?”). Interviews were analysed independently by two authors (JS and SW), who then met to agree their analysis of the pooled phase data (Fig. 1). We subjected the data to interpretative phenomenological analysis, a double hermeneutic process in which the researcher tries to make sense of the participants trying to make sense of their world [19]. Transcripts were merged into a single document and analysed for descriptive, linguistic and normative content before being grouped into higher order themes.

We added phase II to mark the emergence of SD-M as an increasingly important topic during phase I. We recruited HCPs, parents and patients from the same Swiss teaching hospital but used a slightly different approach to the data, Bohnsack’s reconstructive-hermeneutic analysis [20], in a three-step process that takes account of the interplay between parental and HCP viewpoints: first, we analysed the transcripts in terms of each person’s viewpoint in a decision-making context; second, we analysed language use by each participant and its metaphorical content; third, we grouped interviews by the different roles played by patients, parents and HCPs, focusing on the interactions crucial to SD-M. We applied the focus group method in studying the SD-M concept within each participant group.

We then added the ten HCPs in phase III to take account of the fact that although the United States of America (US) is one of the few countries not to have ratified the UNCRC it has a strong impact on the theory and practice of ethical decision-making. We used the same interpretative phenomenological analysis as in phase I.

In the final step we combined the normative UNCRC framework [23] with our descriptive empirical data. Having analysed BIS and SD-M from each viewpoint, we combined the two concepts in a reflective practice approach [21, 22]: Schön’s method frames and solves problematic situations by reflecting not only on past experiences and decisions (reflection-on-action), but also during an ongoing action process (reflection-in-action), involving issues such as the BIS or the UNCRC normative framework; both were observed during the interview process and framed within the shared aim of the UNCRC. Based on participants’ views of the BIS and SD-M and how they interact, we were able to implement a practical reconciliation between the two concepts that we term the shared optimum approach (SOA). We report the results as a synthesis of each phase and the normative UNCRC framework.

## Results

HCPs widely accepted the BIS as a guiding concept in paediatric decision-making, and all participants showed a strong interest in including attitudes from multiple viewpoints in child-centred and family-oriented ways by implicitly combining BIS and SD-M (Table 1).

**Table 1:** Maxims and goal-oriented principles of combining the best interest standard (BIS) with shared decision-making

Topic and themes	Exemplary quotes
Central maxims	
Child-centred	A8: “BIS really means that the child is at the centre, with all its aspects.”
Family-oriented	A5: “We do not have to undertake [medical treatment] just because the family says so and because we respect the family, but the family has been respected as a crucial part of the BIS.”
Goal-oriented principles	
Participation	B22: “There are situations where [our child] is not quite the boss […] but it is basically a sort of a handover of responsibility and decision-making capacity.”
Protection	A12: “You get close to the BIS if a child can be raised without harmful external influences.” B21: Interviewer: “Do you feel that they make too many decision without asking you […]?” Patient: “No, actually no, hmm, they come, and say yes, now, now we just have to do this and then after that, I think that’s good”.
Provision	A5: “The BIS means to be raised and to flourish in a biological, psychological, social and economic environment where all aspects are optimally available.” B25: Interviewer: “What would you make happier [being in a hospital]?” Mother: “Pizza night delivery!” […] Patient: “Yes, or in general, choosing food. At home I love to eat my fries but here it just sucks.” […] Father: “Yes, and what I thought about are the waiting zones. There is no space to wait without face mask, and where you, you know, could sit comfy and drink something”.

### Understanding the BIS

The BIS was used in two ways: narrowly, as a criterion for closed yes/no answers to specific questions (e.g. “[Extracorporeal membrane oxygenation] is not in the best interests of that child”), or broadly, as a normative frame delineating relational responsibilities in recognising the child as the centre of reflection and the family as a precondition for its development (Table S3). We captured BIS-associated goals in three principles: protection, provision and participation (Table 1). Interestingly, participants favouring a narrow interpretation tended to contradict themselves, before switching to a broader interpretation on closer reflection (Table S3).

### Understanding SD-M

SD-M was described neither as an ethical principle nor as a duty to share information but rather as a tool for considering the unique requirements of a child embedded in a family. It was seen as a process of continuously tailored support to implement an optimal plan within constraints delineated by the BIS.

**Fig. 2:**
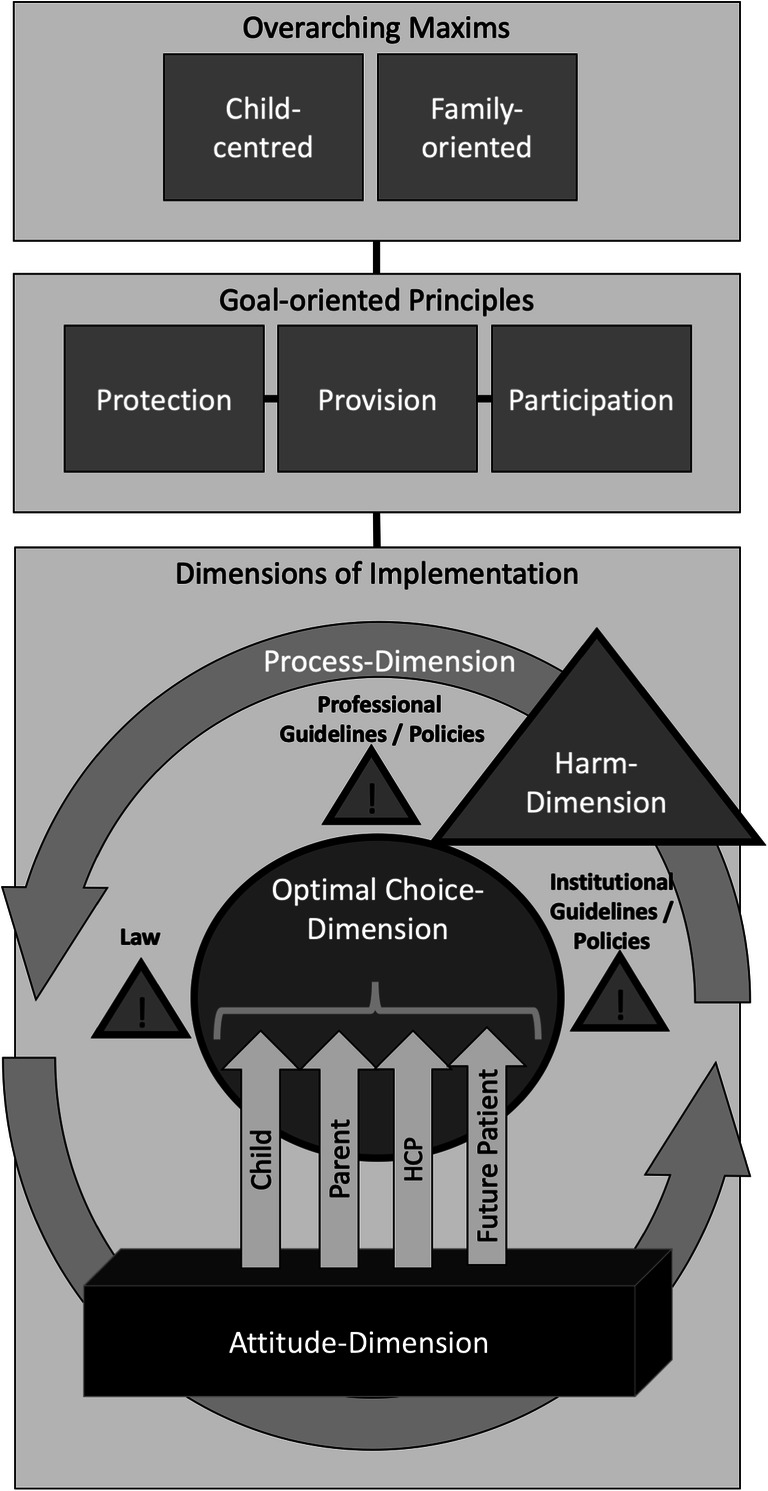
Shared optimum approach reconciling the best interest standard and shared decision-making. HCP, health care professional

Table 3 sketches the various steps in SD-M illustrated by quotes. Steps 1–5—listening and understanding—focus on how a decision is reached and on what values it is based. Steps 6–8—informing, deciding and implementing—address the choices that are feasible within reasonable limits (Fig. 2).

## BIS/SD-M interplay in practice

In our interviews we never based consideration of a child’s well-being and the outcome of specific interventions on the BIS or SD-M alone but always on an amalgam of both. Even constraints on parental decisions in favour of the child’s “best” interests were not set solely on objective medical considerations but also on the specificity of the family and child themselves, both in the present and future (Table S3). The opportunity to mediate between parental and child rights concerning protection, participation and provision appeared the main rationale for reconciling BIS and SD-M.

BIS/SD-M interplay was described in a framework of four complementary dimensions that emerged as a main topic in phase I and were confirmed by the interviews and focus groups in phases II and III (Table 2, Fig. 2):

**Table 2:** The four dimensions of the best interest standard and shared decision-making

Dimension	Exemplary quotes
Attitude	FGB1: “It’s about helping parents to find the best, to find out what they want for their child and what wishes they have.” C36: “Some families have religious objections to the concept of brain death and so may still feel that withdrawing extracorporeal membrane oxygenation is equivalent to killing [their child].” A12: “The social components in which children will grow up vary a lot; you cannot generalise. I think there are so many factors, not just the decision to operate or not, but also the way a child develops.” C40: “Some families, they just want time, it does not matter to them, [...] it’s like they just search for religious faith. No matter what historical, cultural beliefs they have, they feel they need to have that person there, even if that person is just literally only smiling, every now and then, and they love that child, they love that child even until they are in their 30s they love that child.”
Optimal choice	A6: “The BIS aims to support a child’s spiritual, physical and emotional development in an optimal way.” A5: “The maximum is not possible, but one must try to facilitate the optimum of all things feasible.” A12: “How parents cope with a certain problem, how parents communicate a certain problem to their child altogether plays a much more crucial role than simply the decision for or against surgery.” C40: “I used to be against [prolonging life based on cultural beliefs], and now, I’m like, you know, we have the ability to help them and to be with them and that’s what we should do.”
Harm	C31: “The bottom line is, I guess, if it’s thought to be clearly harmful then we are dealing with a situation of medical neglect or abuse essentially and that would call for the legal standard, but at that point […] there would probably be an ethics consult […] to try to resolve the difference in view between families and staff.”
Process	A5: “It’s not about simply the best, it’s about finding something better for this child, [it’s about] a process and about progress.” C33: “Shared decision-making from my perspective is part [of] everything that we do.” B21: Patient: “They tell me what they plan to do and so, and they ask if I can do that and hmm, that’s actually good.” A9: “The interest of a child [in a futile situation] is not to die in a traumatic setting. Therefore, if parents agree, a psychiatrist and the palliative care team should be involved, for not having an emergency but facilitating a process – even if only for some hours or a day, that makes a huge difference for all people involved, the siblings, the family, for everybody.”

### Attitude dimension

All participants emphasised the connection between the BIS and family attitudes to issues relating to medicine, philosophy, culture and religion, describing them as the main source of ethical problems. Attitude recognition is a precondition for combined decision-making.

### Harm dimension

Laws and guidelines set limits on the attitudes of families and HCPs. Not only do they protect children from harm, they also protect them and their carers from unwarranted interference (Table 2, harm).

### Optimal choice dimension

Most interviewees did not believe in optimal choices as such, but rather that a nexus of factors eventually generates choices that are context-dependent and child-centred. Where complex medical decisions were concerned, however, parents and children were not expected to decide independently from HCPs. Based on SD-M steps 1–5, we found strong support for the therapeutic relationship between child, parents and HCPs.

### Process dimension

The fourth dimension comprised the ongoing empowerment of patients and parents in evaluating the decision-making process. It reconciled interviewee interpretations of the BIS and SD-M, breaking down yes/no questions and rigid structures to identify what most needed to be done and who could best facilitate “progress” and “make a huge difference for all people involved, the siblings, the family, for everybody” (Table 2, process).

## The child’s role in decision-making (Table 1)

Recognition that a child’s choice reflects a socio-cultural context was seen as favouring family-oriented paediatric practice. HCPs in particular considered that the BIS actually demands a child-centred approach in order to meet a child’s decision-making needs. The combination of BIS and SD-M mirrored the three Ps of children’s rights [23]: participation, protection and provision. The mother’s understanding of her child being “not quite the boss […]” but accepting “a handover of responsibility and decision-making capacity” showed the two-sided function of capacity as a gatekeeper for autonomy and its role in protecting the child from harmful decisions while empowering participation within a certain harm threshold. The combination of BIS and SD-M appeared ideal for meeting both demands.

## Discussion

Our study highlights the roles of BIS and SD-M in paediatrics and offers a framework for combining the two in ethical decision-making. To our knowledge this is the first empirical study to include both concepts and examine their relationship. Unlike Birchley [1], we found no evidence of substantial inter-concept conflict. Indeed, participant interpretations yielded a child-centred and family-oriented four-dimensional framework with SD-M being applied within the BIS “to facilitate the optimum of all things feasible”, hence our choice of the term “shared optimum approach” (SOA) for combining both concepts.

Within the limits of the BIS, SD-M offers a spectrum between the extremes of a rather paternalistic expert-led style and a patient-/parent-led informed-choice model. Some children, parents or family members show early aptitude in understanding complex facts and reaching largely independent decisions within the SOA, but others need more support.

The SOA separates different tasks (showing respect, limiting harm, defining choices and processes) into separate dimensions (Table 2) and steps (Table 3), mirroring the UNCRC principles of participation, provision and protection [23]. Like the BIS approach proposed by Kopelman [16], the SOA is an umbrella term, allowing stakeholders to adopt positions on various questions in different dimensions, while remaining part of the same “team” aiming at an optimum for the child. Applying the four-dimensional framework counters conceptualisations of children as “incompetent”, “vulnerable” and “passive” recipients of care [24]. At the same time the SOA averts unjustified incursions on family discretion by limiting state intervention according to a harm threshold. But in contrast to the harm standard [12] or the constrained parental autonomy principle [13], the SOA emphasises HCP responsibility within the process dimensions encompassing the support of children and their initially overwhelmed parents. Ideally, the harm threshold is marked out transparently at an early stage, enabling patients, parents and HCPs to focus on achieving the optimum as they navigate difficult situations.

**Table 3:** The eight steps in the shared decision-making process

Step	Exemplary quotes
1) Develop and support partnership/setting	FGB1: “I really try to listen with close attention to adolescents to build a relationship with the message ‘I’m here if you have problems which you’d like to discuss.’” FGB2: “We aim for a good partnership, and sometimes parents need time.”
2) Review information preferences	FGB1: “I do not start to talk, I listen to find out what the child wants to know.” FGB1: “We should not assume that a child can easily talk about highly burdensome information, like upcoming death. For example, I talked with a sibling, who finally said: ‘Why did you tell me all this? I did not want to hear that.’ We need to review and to communicate the reasons for telling something and to reflect also with the parents about the importance [of disclosing information to children] to get the best out of the remaining time. […] Often, I ask the child and the parents separately and frankly where they stand in the process. They are not necessarily at the same point and we have to be very careful [...]. But there are ways to keep the right pace with both parties, reaching a partial consensus at the end.”
3) Review preferred decision-making roles	FGB1: “Regarding a yes or no decision, I remember a father waking up at night in panic, saying ‘I cannot make a decision with saying no for resuscitation.’ […] He did not categorically say no, but just could not consent to let his child die.” FGB2: “I roughly divide parents into those who have a lot of resources and those who initially need more support and help.” FGB2: “I have much respect for natural defence mechanisms [being unable to talk about something]. If nothing comes, I try it with examples, if still nothing comes, then it is just not the right time.” Also see supplemental Table S2
4) Ascertain and respond to ideas, concerns and expectations	C31: “[I’d say to the parents] we want to learn as much as we can from you about what you value and what you think, so that we can make the best decisions for your child.”
5) Identify choices and evaluate evidence from research	FGB2: “Many families are very creative in finding their own choices, which again supports their feeling of being in control, especially if [their choices] evolve from their system and not from outside […]. We need to take care that we find a good way that fits for a family.”
6) Present evidence in an adequate manner; if applicable, define limits.	B22: Parent: “There were one, two situations where I wished that [my daughter] would not have been part of that discussion, for example when they talked about the risk of bleeding, after that she really had sort of a phobia. […] I’d rather discuss certain points with the doctors alone, but after that they always included [my daughter], explaining it in a good way, I think that was important, it did not come from us as parents, but the doctors said ‘These are your parents’ ideas and fears, and we as doctors support that’.”
7) Identify (a) choice(s) within the optimum dimension	B22: Mother: “They said openly that they have little experience [with a specific treatment] [...] they left it relatively open, said to us ‘Take your time, get more information, ask questions.’” Father: “They helped us to get that information which actually was essential in that decision.”
8) Agree on an action plan and follow-up implementation	C33: “I think the shared decision-making from that perspective requires us as providers to reach out to those who have long-term relationships and continuity and may also have a perspective, as well as a relationship with the family outside the hospital.” A6: “We always have to take the consequences of our actions into consideration. Criticism can easily be understood as offensive [...]. But then we should not participate in decision-making at all. The motto rather should be: do not stand still.”

However, in contrast to stand-alone interpretations of the BIS and SD-M, the central task of the SOA is *not* to prioritise the single “best” option for the child or family [25], nor simply to define an impermissible setback to the child’s interests [26], nor even to strive for an equally shared decision. While some have argued that informed consent and assent generally require a minimum level of SD-M [8, 27], the “informed choice” pendulum may have swung too far to the patient side, mostly in the US, requiring that all treatment options be presented to the patient, not just the one the HCP believes is indicated [8, 28]. As we found no significant differences in HCP views of SD-M between our US and Swiss cohorts, we assume that much of the critique against SD-M derives from unjustifiably equating “informed choice” with SD-M. Although children may be decisionally incapable at a given time, their capacity typically evolves and they have a widely acknowledged right to take part in decisions about their care [23, 29]. The paediatric interpretation of SD-M supports this right while also encouraging clinicians to partner with parents. The SOA promotes the sharing of personal and intellectual resources to create a supportive setting that unites participants on a child-centred and family-oriented basis.

That said, the SOA also constrains decision-making in accordance with legal requirements, in particular in the developed world. This is consistent with the view of our international cohort. According to our data, patients, parents and HCPs do not strive for anything “suboptimal”, “just good enough” or “least harmful” [14]. Rather, they strive for an “optimum”, which the Merriam-Webster defines as the “most favourable act to some end […] under implied or specified conditions” [30].

The SOA differs from previous interpretations of the BIS or the isolated harm principle in the change from a one-time decision (e.g. for or against experimental treatment [11]) to a bigger picture over time (i.e. the process dimension). Combining the BIS and SD-M supports a child’s evolving capacity and its relationship within the family during a contested intervention or when deciding palliative care after treatment withdrawal. The parental urge to “do everything” should not be read as medical neglect but as part process within clear limits, creating room for narratives not primarily about prolonging life but about improving its quality. While the attitude dimension creates expectations regarding a specific treatment, the role of the optimal choice and process dimensions is to install child-centred family support, including age-specific knowledge and positive connotations. One cannot foresee how the future will judge current approaches, e.g. managing intersex children. However, in the process dimension, proper documentation of today’s child-centred decision-making will help those who come after us to understand why a decision was once perceived as optimal.

While the SOA cannot simplify ethical complexity, it provides a process that guides analysis and facilitates understanding and decision-making. It also makes for transparent discussion compatible with legal and ethical requirements.

### Limitations

Our conclusions are subject to various limitations and potential biases. First, our data originated from a cohort of patients, parents and HCPs in a Swiss paediatric unit. Despite adequate sample size and control with a US cohort, our results may be insufficiently representative. Second, the semi-structured interview and presentation of clinical issues may have influenced the interviewees; interview fragments and focus on the BIS were shorter than in studies in social science using hermeneutic techniques but comparable to a similar study [5]. Third, two different hermeneutic methods based on the modular three-phase approach may have influenced the results although we reduced potential bias by analysing pooled transcripts. Fourth, although participants widely agreed on how to use both concepts, this cannot be generalised because it was not part of our research question. Fifth, interpretations of best interest may have evolved in the 10 years that have elapsed since the first interview but the addition of more recent interviews provides a preliminary, practice-oriented insight into a hitherto theoretically treated topic. We recently began implementing the SOA tool in ethical case discussions and shall report our critical evaluation in a forthcoming study**.**

## Conclusions

Our empirical study shows that the BIS includes a well-founded harm threshold combined with contextual information based on SD-M. The SOA works as an umbrella concept for the child-centred and family-oriented implementation of the three UNCRC principles within four complementary dimensions. It also works as a response to critiques of the BIS while being a more effective approach to paediatric decision-making than either the BIS or an isolated harm standard. It has four key merits: it structures paediatric decision-making based on the four dimensions starting with known facts and values; it aims for a consensus on various aspects of a harm threshold; this in turn opens up well-defined room for SD-M; finally, it drives a process in which the necessary resources can be identified and established.

## Electronic supplementary material

ESM 1 (DOCX 28 kb)

ESM 2 (DOCX 14 kb)

ESM 3 (DOCX 20 kb)

## Abbreviations

BIS: Best interest standard
HCP: Health care professional
SD-M: Shared decision-making
SOA: Shared optimum approach
UNCRC: United Nations Convention on the Rights of Children
US: United States of America
