# Supplementary material for: Combining the best interest standard with shared decision-making in paediatrics—introducing the shared optimum approach based on a qualitative study
Source: Eur J Pediatr. 2020 Aug 18;180(3):759–66. doi: 10.1007/s00431-020-03756-8 (PMC7886834; doi:10.1007/s00431-020-03756-8)
Supplement: Supplementary file 1 — (DOCX 28 kb) [file 431_2020_3756_MOESM1_ESM.docx]

Supplementary material 1. Consolidated criteria for reporting qualitative research (COREQ): a 32-item checklist for interviews and focus groups according to: Tong, A., Sainsbury, P., & Craig, J. (2007). Consolidated criteria for reporting qualitative research (COREQ): A 32-item checklist for interviews and focus groups. *International Journal for Quality in Health Care*, *19*(6), 349–357. <https://doi.org/10.1093/intqhc/mzm042>

| Domain & Item | Guide question/description | Comments | Reported on page # |
| --- | --- | --- | --- |
| **Domain 1: Research team and reflexivity** | | | |
| *Personal characteristics* | | | |
| 1. Interviewer/facilitator | Which author/s conducted the interview or focus group? | Phase 1: JS/SW  Phase 2: SM/JS  Phase 3: SA/JS | Page 6  Methods |
| 2. Credentials | What were the researcher's credentials? E.g. PhD, MD | One researcher held a PhD, two an MD, three a BA in medicine.  SA, who performed the phase 3 interviews in English, is a US-trained native English speaker. | n/a |
| 3. Occupation | What was their occupation at the time of the study? | DB: sociologist; JS: MD and bioethicist; JJ, SM, SW: medical master student; SA: Student in global healthcare law and bioethics. | n/a |
| 4. Gender | Gender of researcher? | 1 male, 5 female | n/a |
| 5. Experience and training | What experience or training did the researcher have? | Two (DB and JS) had specific qualitative research training and experience. At start JS had been a full-time clinical paediatric resident for 2 years. | n/a |
| *Relationship with participants* | | | |
| 6. Relationship established | Was a relationship established prior to study commencement? | Yes, participants were specifically contacted by recruiters in Phase 1 (JS, SW) and 2 (EB, SM, JS) to determine eligibility, availability and interview location preference. In Phase 3 SA contacted HCPs  in response to snowballing and determined eligibility  and availability by  telephone. | n/a |
| 7. Participant  knowledge of the  interviewer | What did the  participants know  about the researcher?  E.g. personal goals,  reasons for doing the research | Participants knew  the purpose of the study and that it was a doctoral research project with Ethics approval. This information was also provided in  participants’ informed consent document (Phases 1 and 2). Participants in Phase 3 were informed and asked for consent by mail and phone. | n/a |
| 8. Interviewer  characteristics | What characteristics  were reported about  the  interviewer/facilitator?  E.g. bias, assumptions, reasons and interests in the research topic | Participants were informed about JJ, SM and SW being MD candidates and JS being a PhD student and MD with 2 years full-time clinical experience as a paediatric resident; in Phases 2 and 3, they were informed that JS was studying patient, parent and healthcare professional perceptions of best interest and shared decision-making. | n/a |
| **Domain 2: Study design** | | | |
| *Theoretical framework* | | | |
| 9. Methodological orientation and theory | What methodological  orientation was stated  to underpin the study?  E.g. grounded theory,  discourse analysis,  ethnography,  phenomenology,  content analysis | We used hermeneutic methods, interpretative phenomenological analysis (Phases 1 and 3), and Bohnsack’s reconstructive-hermeneutic analysis (Phase 2). | Page 6 Methods |
| 10. Sampling | How were participants  selected? E.g.  purposive, convenience,  consecutive, snowball | Purposive sampling from a range of health professions and families in different age groups was used in Phases 1 and 2, combined with snowballing in Phase 3. | Page 6 Methods |
| 11. Method of  approach | How were participants  approached? E.g. face-to-face, phone, mail, email. | HCPs were contacted by email. Interview details were organised by email and phone. | n/a |
| 12. Sample size | How many  participants were in  the study? | 47 (Table 1). | Page 5 Methods |
| 13. Nonparticipation | How many people  refused to participate  or dropped out?  Reasons? | One dropout in Phase 1 due to change of job. | n/a |
| *Setting* | | | |
| 14. Setting of data  collection | Where was the data  collected? E.g. home,  clinic, workplace | Clinic (Phase 1); home or clinic (Phase 2), depending on family preference and patient situation; phone (Phase 3). | n/a |
| 15. Presence of  non-participants | Was anyone else  present besides the  participants and  researchers? | No. | n/a |
| 16. Description of  sample | What are the  important  characteristics of the  sample? E.g.  demographic data,  date | Figure 1. | Figure 1 |
| *Data collection* | | | |
| 17. Interview  guide | Were questions,  prompts, guides  provided by the  authors? Was it pilot  tested? | We used semi-structured interviews, pilot tested in Phases 1 and 3 but not in Phase 2. Interview guides were semi-structured, participant-led and not strictly followed, especially in Phases 2 and 3. | n/a |
| 18. Repeat  interviews | Were repeat  interviews carried  out? If yes, how  many? | No. | n/a |
| 19. Audio/visual  recording | Did the research use  audio or visual  recording to collect  the data? | Audio only. | Page 5 Methods |
| 20. Field notes | Were field notes made  during and/or after the  interview or focus  group? | SM, JS and SW made field notes during and after the Phase 1 and 2 interviews, but none in Phase 3. | n/a |
| 21. Duration | What was the duration  of the interviews or focus groups? | 45 to 90 minutes. | n/a |
| 22. Data  saturation | Was data saturation  discussed? | Yes. | n/a |
| 23. Transcripts  returned | Were transcripts  returned to  participants for  comment and/or  correction? | Yes in Phase 1, no in Phases 2 and 3. | n/a |
| **Domain 3: Analysis and findings** | | | |
| *Data analysis* | | | |
| 24. Number of  data coders | How many data  coders coded the data? | Phase 1: 2 (JS and SW), Phase 2: 3 (DB, SM, JS)  Phase 3: 2 (JJ, JS) | Page 5 Methods |
| 25. Description of  coding tree | Did authors provide a  description of the  coding tree? | The methods used did not involve a coding tree. | n/a |
| 26. Derivation of  themes | Were themes  identified in advance  or derived from the  data? | The best interest concept was identified in advance as a main theme. Other themes (e.g. shared decision-making) were identified in Phase 1, studied in Phase 2, and combined with best interest in Phase 3. | n/a |
| 27. Software | What software, if  applicable, was used  to manage the data? | Microsoft Excel and Word. | n/a |
| 28. Participant  checking | Did participants  provide feedback on  the findings? | No. | n/a |
| *Reporting* | | | |
| 29. Quotations  presented | \|  \| Were participant quotations presented to illustrate the themes / findings? Was each quotation identified? e.g. participant number \| \| --- \| --- \| | Yes, each referenced to a numbered participant. | Figure 1 and tables; Results |
| 30. Data and  findings  consistent | Was there consistency  between the data  presented and the  findings? | Findings are supported  consistently by interviews and presented by headings and sub-headings in  Results and tables. | n/a |
| 31. Clarity of  major themes | Were major themes  clearly presented in  the findings? | Yes. Major themes are  presented in Results  (e.g. Table 1) and  with reference to the literature in the Discussion. | Page 6-9 Results and tables |
| 32. Clarity of  minor themes | Is there a description  of diverse cases or  discussion of minor  themes? | Yes. Minor sub-themes are discussed as an important part of the resulting shared optimum approach. | Page 8 Results; Table 2; Table S1 |
