# Supplementary material for: Combining the best interest standard with shared decision-making in paediatrics—introducing the shared optimum approach based on a qualitative study
Source: Eur J Pediatr. 2020 Aug 18;180(3):759–66. doi: 10.1007/s00431-020-03756-8 (PMC7886834; doi:10.1007/s00431-020-03756-8)
Supplement: Supplementary file 2 — (DOCX 14 kb) [file 431_2020_3756_MOESM2_ESM.docx]

**Supplementary material**

**Table S2 Narrow closed versus broad process-oriented interpretation**

| Narrow closed | A12: “BIS is approached if a child can be raised without harmful external influences.”  C36: “We obviously don’t offer [a treatment] if it’s not something we feel we can do or would be in the best interest of the child.”  C37: “I think we should do everything we can to keep someone alive and prevent death.” |  | A12: “BI is approached if a child can be raised without harmful external influences.” |
| --- | --- | --- | --- |
| Relational, process-oriented | A12: “The BIS can be highly culture-dependent, interpreted differently in different cultures. The child should be able to develop in a specific society without being impaired in its development and flourishing.”  C36: “I mean in general I see shared decision-making as, um, physicians understanding what a family’s goals and hopes and values are for their child and the things that they want to accomplish and the things that they want to avoid for their child, and then in the context of knowing those values, the physician understands medically what’s happening with the child and can, sort of, make recommendations for care based on knowing those families’ values, um, that’s what I see as good shared decision-making.”  C37: “I don’t know, I don’t know what the right answer is, I think, uh, you know every child is different, every family is different.” |  |  |

| **Table S3 Four perspectives informing the BIS and SD-M** | | | |
| --- | --- | --- | --- |
| **Higher order theme title** | **Exemplary quotes** |  |  |
| a) Perspectives on BIS |  |  |  |
| Parents | A6: “Normally parents and their children are a unit, and parents can act as surrogates for their child in identifying a significant part of its best interests.”  A1: “The parents’ attitude towards a particular treatment has extreme effects on the traumatization of the child. The child can’t be raised normal if parents can’t cope with [the child’s condition].”  A7: “If you think about the best interest of the child in the absence of the family it would make no sense that they would treat one child with a heart transplant and the other not.”  A9: “Winnicott said: ‘There is no such thing as a baby. You can’t care for a baby without taking its primary caregivers into account, namely its parents.” |  |  |
| HCP | A12: “[Parents] may persist in favouring an intervention [genitoplasty] but this does not constitute an indication. The indication can only come from a physician, from a surgeon, finally.” |  |  |
| Child | A9: “In my job, it is crucial to consider the child as a subject. That may sound simple, but in fact it is revolutionary. Even an infant is a subject with particular interests and a will of its own.” |  |  |
| Future being | A1: “Yes, um, well, this [genitoplasty in children with a difference/disorder of sex development] is necessarily a very touchy issue, hotly debated, of course, and very controversially discussed [...] because it is in fact questionable whether these individuals will accept such an intervention retrospectively – especially when something irreversible is taken away.” |  |  |
