# Supplementary material for: Combining the best interest standard with shared decision-making in paediatrics—introducing the shared optimum approach based on a qualitative study
Source: Eur J Pediatr. 2020 Aug 18;180(3):759–66. doi: 10.1007/s00431-020-03756-8 (PMC7886834; doi:10.1007/s00431-020-03756-8)
Supplement: Supplementary file 3 — (DOCX 20 kb) [file 431_2020_3756_MOESM3_ESM.docx]

**Interview guide**

(Streuli, Jürg C., et al. Combining best interest with shared decision-making in paediatrics)

Please note: Interview guides were semi-structured and participant-led. The guides were not strictly followed after the opening questions.

**Phase 1: Focus on best interests^^[[1]](#footnote-1)^^**

Interviews with Health Care Professionals

- Part 1:
  - What does Kindeswohl (or the best interests of the child) mean to you?
  - Are there situations where children are harmed in the context of medical interventions?
  - Can you give us anonymized examples?
  - Can you describe your role in those situations?
  - Can you tell us about the relationship between the well-being of parents and of the child? Is the well-being of parents part of best interests?
- Part 2:
- Case scenario 1: 5-year-old child, leukemia in remission, now referred with newly diagnosed pneumonia. Upon further testing you learn about the relapse of leukemia with metastasis and poor prognosis (confirmed by a second oncologist). While the child gets worse, you discuss intensive care, and part of the team refuses to intubate the child. Parents are overwhelmed by the situation and ask for mechanical ventilation, and even threaten to change hospital if not everything will be done to prolong life.
- Which setting/help would you use immediately or subsequently choose to support decision-making in this situation?
- Which principles and values are relevant for you as a member of the child protection team?
- Case scenario 2: 12-year-old old girl is brought intoxicated to the emergency unit, accompanied by a 16-year-old old friend who found her unconscious behind a public toilet, partially undressed. The friend says that she was on her way to the toilet but did not return. Now the girl is disoriented and unsettled. Because of suspected sexual abuse, the young, still inexperienced resident in charge makes the standard genital examination.
  - Which setting/help would you use immediately or subsequently choose for a decision-making process?
  - Which principles and values are relevant for you as a member of the child protection team?
- Case scenario 3: 2 ½-year-old girl with potential pyelonephritis presents to the emergency department. For a diagnosis, a urinary catheter is needed. Following a first attempt by the resident, the consultant makes three attempts on the screaming, defiant child who has to be restrained by a nurse, a resident, and the father until the catheter is successful.
  - Which setting/help would you use immediately or subsequently choose for a decision-making process?
  - Which principles and values are relevant for you as a member of the child protection team?
- Case scenario 4: A 9-month-old child with difference of sex development (DSD) is presented to the surgical clinic for clitoro- and vaginoplasty. Long-term outcome data on patient satisfaction are non-conclusive and inconsistent. Surgery is strongly wished by the parents. The mother says that she can hardly change the diaper of her own child and has had psychological help. An interdisciplinary team argues that surgery might be in the best interest, based on the strong psychological strain on the mother.
  - Which setting/help would you use immediately or subsequently choose for a decision-making process?
  - Which principles and values are relevant for you as a member of the child protection team?
- Case scenario 5: Parents would like to use the kidney of their 4-year-old son for his sister. They argue that it would be an altruistic act from their son and that it would potentially be traumatizing for him if his sister were to die.
  - Which setting/help would you use immediately or subsequently choose for a decision-making process?
  - Which principles and values are relevant for you as a member of the child protection team?
- General questions: What could one currently do to support child-oriented care?
- Do you have anything else to add?
- Closing and expression of thanks.

**Phase 2: Focus on shared decision-making (translated from German)**

Focus groups with health care professionals

- Input statement: I start with the following quote from Kierkegaard: “In order to help someone, we must first and foremost understand where he or she stands.” In the next 45 minutes, I’d like to learn from you how you can understand your paediatric patients. Maybe you could start by giving an example of how you explore where your patient stands.
- Part II: How would you describe in general the communication between you and the child?
- Part III: We reached the third part of the interview. Ten years ago one of the first articles on shared decision-making in pediatrics by Bauchner was published, summarizing the most important points as follow:
  - Develop a partnership with the patient
  - Review the patient’s preference for information
  - Review the patient’s preference for role in decision-making
  - Ascertain and respond to patient’s ideas, concerns, and expectations
  - Identify choices and evaluate evidence from research
  - Present evidence
  - Make or negotiate a decision
  - Agree on an action plan.
  - How well these principles will work

In this last part I would like to hear your opinion, how you could or do implement these points.

- Do you have anything to add?
- Closing and expression of thanks.

Interviews with Families

- **General Situation:** Would you like to tell us why you are treated at the children’s hospital?
- **Communication in general:**
  - How would you describe the communication between doctors, the child, and the parents? What are your experiences?
  - What has been positive/negative?
  - What role in communication did you play?
- **Decision-making in general:**
  - How do you think decisions were made?
  - Could you give us examples?
  - How could the decision-making process be improved?
  - Can you describe if and why any dissatisfying decisions have been made?
- **Decision-making more detailed:**
  - Did you have wishes or preferences which should have been assessed earlier?
  - Would it have been useful to note your/your child’s wishes or opinions in advance, prior to a conversation with doctors?
  - Could you name five wishes or opinions which are relevant for you?
  - Were there questions or decisions which should have been deferred?
- **Decision-making tools**
  - Which tools were offered and/or used?
  - Which tools did you wish would have been used?
  - What should an online tool look like, and would you use it?
  - (if not asked to the child directly)…and would you (the child) use it?
  - Would you give as five issues, which probably are hardly asked, but would be of importance (if the HCPs knew about it)
- Debriefing
  - Are there important aspects which were not mentioned?
  - How was this interview for you?

**Phase 3: Focus on best interests and shared decision-making (USA) ^^[[2]](#footnote-2)^^**

Interviews with healthcare professionals

- Can you tell me about your views on shared decision making in pediatrics?
- How does it come up in your practice?
- How is this term used?
- What are the challenges and problems that come up in making a shared decision?
- To what extent is the family involved in making the decisions?
- How do you define best interest? And how do you work towards finding it?
- How do you see the relationship between the parents’ and the child’s best interest?
- What do you refer to, to help in making a decision?

1. Only questions focusing on BIS/SD-M are shown. A part of the interview was on child protection services. [↑](#footnote-ref-1)
2. Only questions focusing on BIS/SD-M are shown. A part of the interview was on the ethics of extracorporeal membrane oxygenation in pediatrics. [↑](#footnote-ref-2)
